# Disentangling the Effects of Processing Speed on the Association between Age Differences and Fluid Intelligence

**DOI:** 10.3390/jintelligence8010001

**Published:** 2019-12-25

**Authors:** Anna-Lena Schubert, Dirk Hagemann, Christoph Löffler, Gidon T. Frischkorn

**Affiliations:** 1 Institute of Psychology, Heidelberg University, Hauptstrasse 47-51, D-69117 Heidelberg, Germany; dirk.hagemann@psychologie.uni-heidelberg.de (D.H.); Christoph.Loeffler@psychologie.uni-heidelberg.de (C.L.); 2 Department of Psychology, University of Zurich, Binzmuehlestrasse 14, 8050 Zurich, Switzerland; gidon.frischkorn@psychologie.uzh.ch

**Keywords:** intelligence, aging, mental speed, reaction time, diffusion model, EEG, ERP

## Abstract

Several studies have demonstrated that individual differences in processing speed fully mediate the association between age and intelligence, whereas the association between processing speed and intelligence cannot be explained by age differences. Because measures of processing speed reflect a plethora of cognitive and motivational processes, it cannot be determined which specific processes give rise to this mediation effect. This makes it hard to decide whether these processes should be conceived of as a cause or an indicator of cognitive aging. In the present study, we addressed this question by using a neurocognitive psychometrics approach to decompose the association between age differences and fluid intelligence. Reanalyzing data from two previously published datasets containing 223 participants between 18 and 61 years, we investigated whether individual differences in diffusion model parameters and in ERP latencies associated with higher-order attentional processing explained the association between age differences and fluid intelligence. We demonstrate that individual differences in the speed of non-decisional processes such as encoding, response preparation, and response execution, and individual differences in latencies of ERP components associated with higher-order cognitive processes explained the negative association between age differences and fluid intelligence. Because both parameters jointly accounted for the association between age differences and fluid intelligence, age-related differences in both parameters may reflect age-related differences in anterior brain regions associated with response planning that are prone to be affected by age-related changes. Conversely, age differences did not account for the association between processing speed and fluid intelligence. Our results suggest that the relationship between age differences and fluid intelligence is multifactorially determined.

## 1. Introduction

Age-related changes in cognitive abilities across the lifespan follow a multi-directional pattern (Baltes 1987). Fluid intelligence tends to gradually decline from young or middle adulthood (Horn and Cattell 1967; Nettelbeck and Burns 2010; Salthouse 2004), although a recent study demonstrated substantial heterogeneity in the timing of developmental peaks and the subsequent decline of different fluid abilities (Hartshorne and Germine 2015). In contrast, crystallized intelligence tends to increase across the lifespan (Horn and Cattell 1967; Salthouse 2004), reflecting knowledge and abilities accumulated throughout life that remain relatively stable until late life and may be used to compensate losses in fluid abilities (Hedden et al. 2005; Hedden and Gabrieli 2004).

Factor-analytical studies suggest that a large part of fluid cognitive abilities declines in concert (Salthouse and Ferrer-Caja 2003; Salthouse 2004; Tucker-Drob 2011), suggesting that their joint decrease may be driven by developmental changes in either a single or many interacting elementary processes. In cross-sectional studies, the negative association between age and fluid intelligence can be broken down into a large negative association between age and the general factor of fluid intelligence and moderate negative associations between age and the lower-level factors of processing speed and memory (Salthouse and Ferrer-Caja 2003; Salthouse 2004). Results are similar in longitudinal studies, where a general factor of participants’ aging slopes across different cognitive domains accounted for 39 percent of the variance in change in any given variable (Rhemtulla and Tucker-Drob 2011; Tucker-Drob 2011; Tucker-Drob et al. 2014). In addition, there are also task- and domain-specific trajectories of cognitive aging (Hartshorne and Germine 2015; Tucker-Drob 2011), supporting the idea that cognitive aging should be conceived of as a hierarchical construct. Given the fact that a large proportion of age-related cognitive decline can be explained by a general factor of cognitive aging, it is still important to identify elementary cognitive processes that show a comparable age-related decline and may affect a broad range of cognitive abilities.

### 1.1. The Role of Processing Speed in Cognitive Aging

The age-related slowing of processing speed has long been suggested to drive age-related changes in cognitive abilities (Birren and Fisher 1995; Finkel et al. 2007; Salthouse 1985 1996). Younger adults show a substantially higher processing than older adults (Hartshorne and Germine 2015; Salthouse 2009). Yet, data from longitudinal studies suggest that this age difference may have been overestimated due to cohort effects (Schaie 2005).

Most intriguingly, individual differences in processing speed have often been found to substantially mediate the relationship between age and fluid intelligence both in cross-sectional (Salthouse 1996) and in longitudinal studies (Finkel et al. 2007; Robitaille et al. 2013). However, some studies failed to find evidence for this mediation (Deary et al. 2009) or found that the amount of shared variance was significantly smaller in longitudinal in comparison to cross-sectional studies (Lindenberger et al. 2011; Zimprich and Martin 2002). In a meta-analysis of 91 studies, Verhaeghen and Salthouse (1997) estimated that 71 to 79 percent of the age-related variance in cognitive abilities was shared with processing speed, suggesting that a large proportion of the age-related variance in a wide range of cognitive ability measures is shared with measures of processing speed. Conversely, age does not seem to account for much of the covariance between processing speed and intelligence (Bors and Forrin 1995), indicating that the association between processing speed and intelligence is invariant across the lifespan.

There is, however, an ongoing debate whether the age-related slowing of processing speed should be considered the cause or an early symptom of age-related cognitive decline. The former hypothesis is based on the idea that individual differences in processing speed may affect the efficiency of information-processing by affecting the signal-to-noise ratio or the amount of information that can be simultaneously held in working memory (Jensen 2006; Salthouse 1996). The latter hypothesis presumes that age-related slowing reflects age-related neural degeneration (Birren 1974) or even the aggregate effect of many small, independent age-related changes in the nervous system (Hartley 2006) that adversely affect higher-order cognitive abilities.

Despite the important role of processing speed, there are two challenges to the notion that the age-related slowing of processing speed drives age-related changes in intelligence. First, properties of other processes such as working memory capacity and sensory acuity also share a substantial amount of variance with the age-related variance in intelligence (Anstey et al. 2001; Salthouse 2001), suggesting that processing speed is not the only variable showing a near-parallel pattern of change and a substantial association with changes in cognitive abilities. Second, processing speed is typically measured as mean reaction times in experimental tasks or as performance in clerical speed tests (Verhaeghen and Salthouse 1997). Performance in both kinds of tasks is affected by a number of different processes including sensory processing, encoding, evidence accumulation, response preparation, and response execution, and may be additionally affected by strategic or motivational considerations reflected in differing speed-accuracy tradeoffs. Taken together, these issues amount to questions about the validity of processing speed measures: As long as it is unknown which of many conceivable processes contribute to the association between processing speed and cognitive aging, it cannot be determined if age-related slowing should be conceived of as a cause or an indicator of cognitive aging.

### 1.2. A Neurocognitive Psychometrics Account of Mental Speed

In the present paper, we want to address this issue by using a neurocognitive psychometrics account of mental speed to decompose the association between age differences and fluid intelligence and shed light on the question whether the processes giving rise to the association between age differences and fluid intelligence can be conceived of as a cause or an indicator of cognitive aging. For this purpose, we employed diffusion modeling and chronometric analyses of the event-related potential (ERP) of the electroencephalogram (EEG) to identify specific age-related processes in information-processing and decision-making.

#### 1.2.1. Diffusion Modeling

**Figure 1:**
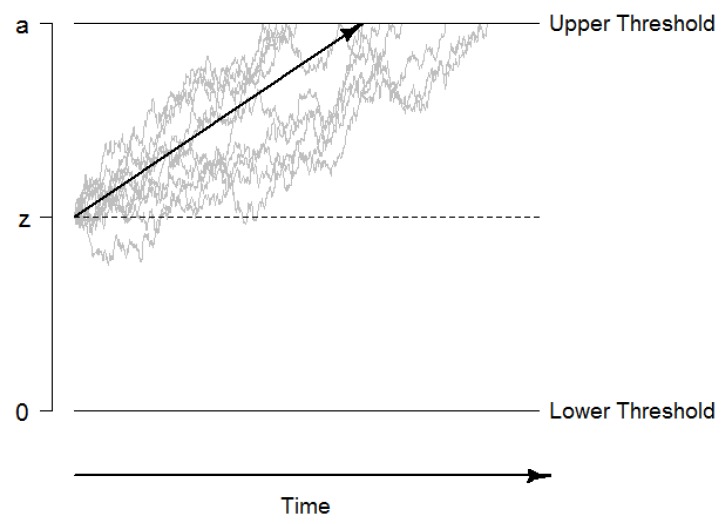
Graphical illustration of the diffusion model. The decision process starts at the starting point *z*, and information is accumulated until the boundary *a* is reached. The systematic part of the accumulation process, the drift rate *v*, is illustrated with the black arrow. The non-decision time $t_{0}$ is not included in this figure.

The diffusion model is a validated mathematical model that describes decision making as a random-walk evidence accumulation process (see Figure 1), which continues with an average evidence accumulation rate *v* and a fixed instantaneous variance until one of two decision thresholds is reached (Ratcliff 1978; Voss et al. 2004). The model takes into account the whole response time distribution of both correct and incorrect responses and uses these distributions to estimate at least four parameters. The drift rate *v* reflects the average evidence accumulation rate of the random-walk process and can be considered a measure of the speed of information uptake. The boundary separation *a* reflects a predetermined decision criterion and accounts for person-specific or experimentally induced speed-accuracy tradeoffs. The starting point *z* reflects an a priori bias in decision making in favor for one of the two alternatives. Finally, the non-decision time $t_{0}$ is a composite of the time needed for all non-decisional processes such as sensory processing, encoding, and response execution.

Because the diffusion model provides a process-based account of decision making that allows the measurement and mathematical separation of different processes involved in decision making, it has become increasingly popular in individual differences and aging research (e.g., Dirk et al. 2017; Dully et al. 2018; Frischkorn and Schubert 2018; Huff and Aschenbrenner 2018; Nunez et al. 2015; Ratcliff et al. 2003; Schubert et al. 2015, 2016, 2019; Schmiedek et al. 2007; Schmitz and Wilhelm 2016; Schmitz et al. 2018; Spaniol et al. 2008; Yap et al. 2012; Ratcliff et al. 2004; Ratcliff et al. 2010; Ratcliff et al. 2011). The drift rate parameter in particular has been consistently associated with intelligence (Ratcliff et al. 2010, 2011; Schmiedek et al. 2007; Schmitz and Wilhelm 2016; Schmitz et al. 2018; Schubert et al. 2015; Schubert et al. 2019), suggesting that smarter individuals benefit from a higher rate of evidence accumulation. In comparison, there is no consistent evidence that more intelligent individuals also show shifts in boundary separation or shorter non-decision times in comparison to less intelligent individuals (but see, Ratcliff et al. 2010; Ratcliff et al. 2011; Schmiedek et al. 2007).

Results are more heterogeneous in the realm of cognitive aging, where associations with age differences have been reported for all three parameters. In a recent review, Dully et al. (2018) summarized evidence from 14 studies demonstrating that older adults showed higher decision boundaries, suggesting that at least part of the association between age and reaction times might be due to a (strategic or involuntary) increase in decision cautiousness. Moreover, they reported that older adults also showed consistently longer non-decision times across eight studies, indicating that some amount of the association between age and reaction times can be explained by individual differences in encoding and/or motor response speed. Finally, they stated that the evidence regarding associations between age and drift rates was less conclusive, as these associations were largely task-dependent. A recent meta-analysis suggested that the association between age differences and drift rates may be moderated by task content, with older adults showing lower drift rates in perceptual and memory tasks and higher drift rates in lexical decision tasks (Theisen et al. 2019). This task-dependency of age-related associations in drift rates is surprising, as drift rates are considered to be the one diffusion model parameter to show substantial consistency across tasks (Schubert et al. 2016), but may be reconciled with the fact that fluid and crystallized abilities typically show opposing aging trajectories (Schaie 2005). Therefore, it is important to keep in mind that drift rates do not reflect a person’s general rate of evidence accumulation, but their rate of evidence accumulation in a specific task (Frischkorn and Schubert 2018).

Taken together, individual differences in intelligence have been shown to be consistently related to drift rates, whereas age differences have been shown to be associated with boundary separation, non-decision time, and drift rate parameters. Because age-related associations can be observed in several diffusion model parameters that each affects mean reaction times, it is important to analyze which—if any—of these processes mediates the association between age differences and fluid intelligence. If a large part of the mediating effect of processing speed can be attributed to individual differences in decision criteria and/or non-decision times, criticisms of the validity of processing speed as a mediator of age-related cognitive changes are justified. Nevertheless, even if only a relatively small amount of the association between age differences and reaction times can be attributed to individual differences in drift rates, the age-related variance in drift rates may still explain a substantial amount of the age-related variance in cognitive abilities. In the present study, we therefore investigated whether individual differences in diffusion model parameters account for any age-related variance in cognitive abilities.

#### 1.2.2. Chronometric Analyses of the ERP

The ERP allows to decompose the electrophysiological activity between stimulus onset and response into functionally distinct components associated with specific cognitive processes. The latencies of these components can be regarded as measures of processing speed of the associated processes, with shorter latencies reflecting a higher speed of the respective process associated with a specific ERP component. While fluid intelligence has been shown to be specifically associated with latencies of ERP components reflecting higher-order cognitive processes such as stimulus evaluation, attention, memory updating, and response selection (i.e., latencies of the N2 and P3 component, Bazana and Stelmack 2002; McGarry-Roberts et al. 1992; Schubert et al. 2017; Troche et al. 2009, 2015), associations with age have been found both for latencies of ERP components associated with earlier processing such as the P1 and N1 and for latencies of ERP components associated with higher-order processing such as the N2 and P3 (De Sanctis et al. 2008; Friedman 2012; Gajewski et al. 2018; Hämmerer et al. 2014; Polich 1996; Price et al. 2017; Schapkin et al. 2014).

The results regarding age differences and early processing are, however, not entirely consistent: While some studies found evidence for slower visual P1 and N1 components in older adults, other studies failed to find any difference in visual P1 and N1 latencies between age groups (De Sanctis et al. 2008; Gajewski et al. 2018), suggesting that any effects of age on the speed of earlier visual processing are comparatively small. Moreover, age differences in early sensory processing were not observed in the auditory domain (Friedman 2012). In comparison, age differences in latencies of the N2 and P3 component are well-documented (Friedman 2012; Gajewski et al. 2018; Hämmerer et al. 2014; Polich 1996; Price et al. 2017; Schapkin et al. 2014), indicating that older adults show slower higher-order cognitive and attentional processes, as both ERP components have been associated with attentional processes such as memory updating (Polich 2007), cognitive control (Folstein and Van Petten 2008), and attention (Folstein and Van Petten 2008).

Taken together, both fluid intelligence and age differences are substantially related to latencies of ERP components associated with higher-order cognitive processes, suggesting that the same neural mechanisms underlying age-independent variation in cognitive abilities may also underlie age-dependent variation. In the present study, we will therefore investigate whether individual differences in these ERP latencies account for any age-related variance in cognitive abilities.

### 1.3. The Present Study

The aim of the present study was to identify which processes-related parameter may explain the fact that individual differences in processing speed fully mediate the association between age differences and fluid intelligence. For this purpose, we used a neurocognitive psychometrics account of mental speed to decompose the association between age differences and fluid intelligence. Reanalyzing data from two previously published datasets (Frischkorn et al. 2019; Schubert et al. 2017), we investigated whether individual differences in diffusion model parameters and in ERP latencies associated with higher-order attentional processing explained the negative association between age differences and fluid intelligence. Moreover, we investigated if the phenomenon that age does not account for the association between processing speed and intelligence can be generalized to electrophysiological and model-based measures of processing speed.

## 2. Materials and Methods

The data included in the present study were collected in two research projects on the neurocognitive correlates of intelligence and merged across studies to increase the stability of statistical estimates. EEG data were only available for one of the two projects (see below).

### 2.1. Participants

We collected data from N=223 (125 females, 98 males) participants from different occupational and educational backgrounds in the Rhine-Neckar metropolitan region, Germany. Participants were between 18 and 61 years old ($M_{age}=37.9$, $Med_{age}=41$, $SD_{age}=14.1$), had normal or corrected to normal vision, and reported no history of mental illness. All participants signed an informed consent prior to their participation in the study.

### 2.2. Procedure

The data from Schubert et al. (2017) were collected at two sessions approximately four months apart. Participants completed the experimental tasks during the first session and the cognitive ability tests at the second session. During the first session, they were seated in a dimly-lit, sound-attenuated cabin while their EEG was recorded. The second session took place in groups of up to four participants. All participants completed the experimental tasks and cognitive ability tests in the same order. Each session lasted about 3–3.5 h.

The data from Frischkorn et al. (2019) were collected during a single session lasting about 3 h in groups of up to four participants. All participants completed the experimental tasks and cognitive ability tests in the same order.

### 2.3. Measures

#### 2.3.1. Experimental Tasks

Participants completed the Sternberg memory scanning task and the Posner letter matching task.

The *Sternberg memory scanning task* consisted of two conditions with different memory set sizes (three and five). In each condition, participants saw a memory set of three (S3) or five (S5) sequentially presented white digits on a black screen. Each digit was presented for 1000 ms, followed by a blank inter-stimulus interval shown for 400–600 ms. The final digit was followed by a white question mark shown on a black screen for 1800–2200 ms, after which a single white digit was shown as the memory probe. The probe digit was included in the memory set in 50% of the trials. Each trial was followed by a uniformly distributed ITI of 1000–1500 ms. In the study by Schubert et al. (2017), conditions were presented blockwise with 10 practice and 100 experimental trials each and the order of conditions was counterbalanced across participants. In the study by Frischkorn et al. (2019), conditions were presented blockwise with 10 practice and 40 experimental trials each and the order of conditions was kept constant across participants.

The *Posner letter matching task* consisted of two conditions: The physical identity (PI) condition, in which participants had to decide whether two letters were physically identical, and the name identity (NI) condition, in which participants had to decide whether two letters were semantically identical. Letters were identical in 50% of the trials. In each condition, participants saw two white letters on a black screen that remained unchanged for 1000 ms after their response. Each trial was followed by an inter-trial interval (ITI) of 1000–1500 ms. Conditions were presented blockwise and the order of conditions (PI first) was kept constant across participants. In the study by Schubert et al. (2017), participants completed 10 practice and 300 experimental trials of each condition, whereas in the study by Frischkorn et al. (2019), participants completed 10 practice and 40 experimental trials of each condition.

#### 2.3.2. Intelligence Test

Fluid intelligence was assessed with the short version of the Berlin Intelligence Structure test (BIS; Jäger and Süß 1997), which is a fluid intelligence test that distinguishes between four operation-related (processing speed, memory, creativity, processing capacity), and three content-related (verbal, numerical, figural) components of cognitive abilities (see Bucik and Neubauer 1996, for an independent validation of the factor structure). Each subtest consists of a combination of one operation-related with one content-related component. Following the manual, we calculated participants’ scores in the four operation-related components by aggregating the normalized *z*-scores of tasks reflecting the specific operational components irrespective of content. The mean score of the processing capacity (PC) component was M=99.73(SD=10.35), the mean score of the processing speed (PS) component was M=96.49(SD=10.80), the mean score of the memory (M) component was M=97.49(SD=8.48), and the mean score of the creativity (C) component was M=98.10(SD=10.38). We then transformed these scores to *z*-scores for further analyses.

#### 2.3.3. EEG Recording

EEG data during the two experimental tasks were only recorded in the study by Schubert et al. (2017). Hence, the EEG data set consists of a subsample of N=122 participants (72 females, 50 males, $M_{age}=36.73$, $Med_{age}=35$, $SD_{age}=13.58$).

Participants’ EEG was recorded with 32 equidistant Ag/AgCl electrodes, a 32-channel BrainAmp DC amplifier (Brain Products, Munich) and a sampling rate of 1000 Hz (software bandpass filter of 0.1–100 Hz with a slope of 12 db/octave). In addition, we recorded participants’ electrooculogram (EOG) bipolarly with two electrodes positioned above and below the left eye and two electrodes positioned at the outer canthi of the eyes. Electrode impedances were kept below 5 kΩ. Data were collected with a central electrode reference and offline re-referenced to the average activity of all electrodes (average reference). The data were filtered offline with a low-pass filter of 16 Hz and a slope of 12 db/octave.

### 2.4. Data Analysis

#### 2.4.1. Reaction Time Data

We removed intraindividual outliers separately for each task and each condition. For this purpose, we first discarded any incorrect responses and any responses faster than 150 ms or slower than 3000 ms. We then discarded for each participant any trials with logarithmized RTs exceeding ±3 SDs from their respective condition means. Subsequently, we calculated participants’ mean RTs for each condition and transformed them to *z*-scores for further analyses.

Diffusion model parameters were estimated with fast-dm-30.2 (Voss and Voss 2007) using the maximum likelihood criterion for optimization. Parameters were estimated separately for each participant and each experimental condition. We estimated participants’ drift rate (*v*), boundary separation (*a*), non-decision time ($t_{0}$), and inter-trial variability of the non-decision time ($s_{t0}$). The starting point *z* was fixed to the center between of two decision thresholds. All other parameter were fixed to zero. These model estimation settings follow the recommendations by Lerche and Voss (2016).

#### 2.4.2. Electrophysiological Data

ERPs were calculated separately for each task and condition by averaging the electrophysiological activity time-locked to the onset of the response stimulus. Epochs had a duration of 1200 ms, including a baseline of 200 ms prior to stimulus onset. Ocular artifacts were corrected for with the regression procedure suggested by Gratton et al. (1983). We discarded epochs with amplitudes exceeding ±70 μV at least once within the time window, with amplitude changes exceeding 100 μV within 100 ms, or with activity lower than 0.5 μV within 100 ms as artifacts.

For each participant, latencies of three ERP components previously shown to be associated with intelligence (P2, N2, P3) were determined. We determined the P2 peak latency at the fronto-central electrode over midline, and the N2 and P3 latencies at the parietal electrode over midline (see Schubert et al. 2017 for grand averages of the experimental tasks). Peak latencies were determined separately for each condition of each experimental task. Subsequently, peak latencies exceeding ±3 SDs from the mean peak latency of each condition were discarded and the remaining peak latencies were transformed to *z*-scores for further analyses.

#### 2.4.3. Statistical Analyses

We used structural equation models to assess the associations between age, fluid intelligence, reaction times, ERP latencies, and diffusion model parameters. We first investigated the association between age, fluid intelligence, and reaction times. We specified a mediation model to replicate the finding that reaction times mediate the association between age and fluid intelligence in our data. We also tested whether we could replicate the reverse finding, i.e. that age differences do not account for the association between reaction times and fluid intelligence, in our data. We then specified a second set of models, in which we used all measures aimed to decompose the stream on information-processing into process-related measures (i.e., diffusion model parameters and ERP latencies) and analyzed their relation to age differences and fluid intelligence. In this set of models, we also investigated which process-related measures contributed to the association between age differences and fluid intelligence by including them simultaneously in a mediation model. Due to the task-specificity of diffusion model parameters, residual correlations between model parameters were allowed within conditions of each experimental task. All significant mediators were subsequently entered into a hierarchical mediation model to explore if they accounted jointly or independently for the association between age differences and fluid intelligence. Finally, we explored in a third set of models if age differences accounted for any association between process-related variables and fluid intelligence.

All models were fitted with the *R* package *lavaan* (Rosseel 2012) with the maximum likelihood algorithm with robust Huber-White standard errors and a scaled test statistic equal to the Yuan-Bentler test statistic to account for the non-normality of some measures. We evaluated goodness-of-fit with the comparative fit index (CFI; Bentler 1990) and the root mean square error of approximation (RMSEA; Browne and Cudeck 1992). Following the recommendations by (Browne and Cudeck 1992; Hu and Bentler 1999), we considered CFI values > 0.90 and RMSEA values < 0.08 to indicate acceptable model fit, and CFI values > 0.95 and RMSEA values < 0.06 to indicate good model fit.

Data and analysis code are available at https://osf.io/hy5fw/.

## 3. Results

### 3.1. Associations between aGe Differences, Fluid Intelligence, and Reaction Times

Mean accuracies and reaction times for each condition of the two experimental tasks are shown in Table 1.

**Figure 2:**
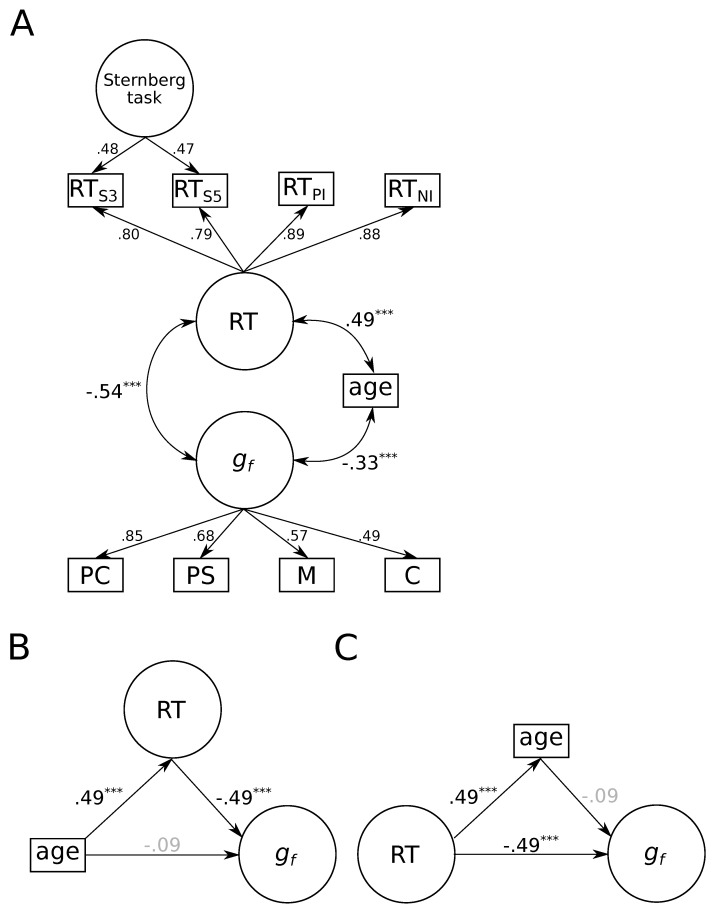
Graphical illustration of three structural equation models on the association between age, fluid intelligence, and reaction times. (**A**) Correlations between age differences, fluid intelligence, and reaction times. (**B**) Mediation of the association between age differences and fluid intelligence by reaction times. (**C**) Mediation of the association between reaction times and fluid intelligence by age differences. Standardized regression weights and correlation coefficients are shown next to paths with non-significant associations shaded in gray. RT = reaction time; $g_{f}$ = fluid intelligence; S3 = Sternberg task, memory set 3; S5 = Sternberg task, memory set 5; PI = Posner letter matching task, physical identity; NI = Posner letter matching task, name identity; PC = processing capacity; PS = processing speed; M = memory; C = creativity. N=223. *** *p* < 0.001.

The corresponding structural equation model (see Figure 2A) provided a good account of the data, $\chi^{2}\left(36\right)=66.27$, p=0.002, CFI=0.96, RMSEA=0.06, 95% CI = [0.04; 0.08]. As expected, older participants showed slower RTs, r=0.49, p<0.001, 95% CI = [0.39; 0.59], and lower fluid intelligence, r=−0.33, p<0.001, 95% CI = [−0.46; −0.20]. Moreover, there was a negative correlation between fluid intelligence and RTs, r=−0.54, p<0.001, 95% CI = [−0.67; −0.41].

To evaluate if we could replicate the finding that the negative correlation between age and fluid intelligence could be accounted for by individual differences in reaction times, we constructed a second model in which individual differences in reaction times mediated the correlation between age and fluid intelligence (see Figure 2B, p. 9). The fit of this model was identical to the first model. We found that the association between age and fluid intelligence was fully mediated by individual differences in reaction times as indicated by the non-significant direct effect, β=−0.09, p=0.262, 95% CI = [−0.25; 0.07], and the significant indirect effect, β=−0.24, p<0.001, 95% CI = [−0.34; −0.14]. About 72% of the association between age and fluid intelligence was accounted for by individual differences in reaction times. Subsequently fixing the direct path to zero did not impair model fit, $\chi_{LLR}^{2}\left(1\right)=1.23$, p=0.267, suggesting that the association between age differences and fluid intelligence was fully mediated by individual differences in reaction times.

Conversely, age did not mediate the association between reaction times and fluid intelligence (see Figure 2C, p. 9), as indicated by the insignificant indirect effect, β=−0.05, p=0.257, 95% CI = [−0.12; 0.03]. These results are consistent with the finding by Bors and Forrin (1995) that individual differences in reaction times fully account for the association between age differences and intelligence, whereas age differences do not account for the correlation between reaction times and intelligence.

### 3.2. Which Process-Related Parameter Accounts for the Association between Age Differences and Fluid Intelligence?

We then explored which process-related parameters might account for the association between age differences and fluid intelligence. Mean diffusion model parameter estimates and ERP latencies for each condition of the two experimental tasks are shown in Table 1. The estimated diffusion model parameters provided an excellent account of the empirical data, with correlations between empirical and predicted mean RTs ranging from r=0.94 to r=1.00 and correlations between empirical and predicted accuracies ranging from r=0.72 to r=0.89.

**Figure 3:**
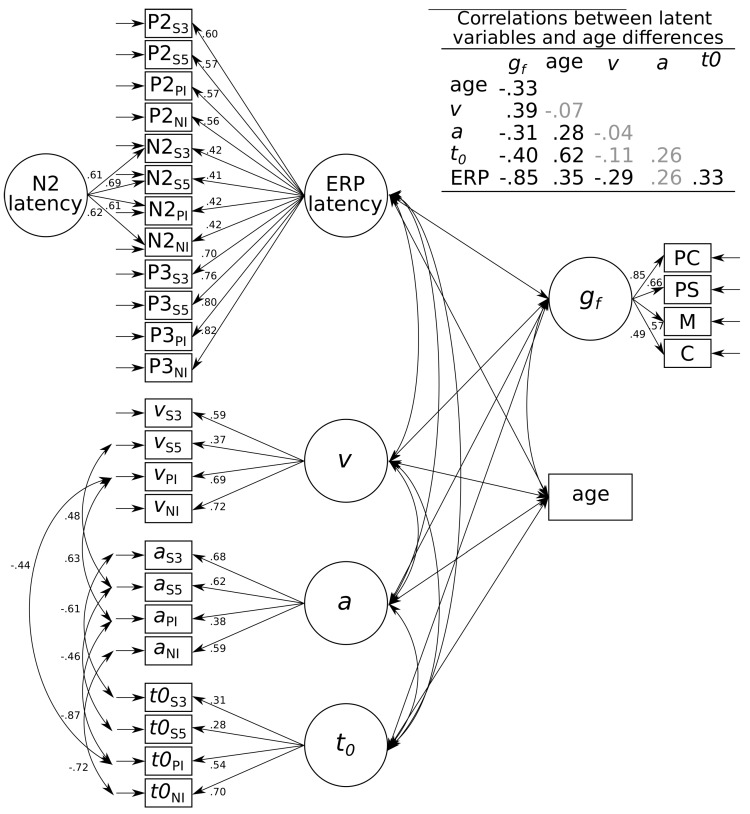
Graphical illustration of the associations between age differences, fluid intelligence, diffusion model parameters, and ERP latencies. Standardized residual correlation coefficients are shown next to paths, whereas standardized correlations between latent variables and age differences are shown in the table in the upper right corner with non-significant correlations shaded in grey. *v* = drift rate; *a* = boundary separation; $t_{0}$ = non-decision time; ERP = event-related potential; $g_{f}$ = fluid intelligence; S3 = Sternberg task, memory set 3; S5 = Sternberg task, memory set 5; PI = Posner letter matching task, physical identity; NI = Posner letter matching task, name identity; PC = processing capacity; PS = processing speed; M = memory; C = creativity. N=223.

We first explored how age and fluid intelligence correlated with all process-related parameters (i.e., diffusion model parameters and ERP latencies). The model (see Figure 3, p. 11) provided an acceptable account of the data, $\chi^{2}\left(388\right)=685.42$, p<0.001, CFI=0.83, RMSEA=0.06, 95% CI = [0.05; 0.07]. Older adults showed higher boundary-separations, r=0.28, p=0.009, 95% CI = [0.13; 0.44], slower non-decision times, r=0.62, p<0.001, 95% CI = [0.50; 0.75], and slower ERP latencies, r=0.35, p<0.001, 95% CI = [0.20; 0.49], but no differences in drift rates, r=−0.07, p=0.402, 95% CI = [−0.23; 0.09]. Conversely, more intelligent individuals showed higher drift rates, r=0.39, p=0.002, 95% CI = [0.20; 0.58], lower boundary-separations, r=−0.31, p=0.005, 95% CI = [−0.48; −0.15], faster non-decision times, r=−0.40, p=0.001, 95% CI = [−0.58; −0.22], and faster ERP latencies, r=−0.85, p<0.001, 95% CI = [−0.96; −0.74].

**Figure 4:**
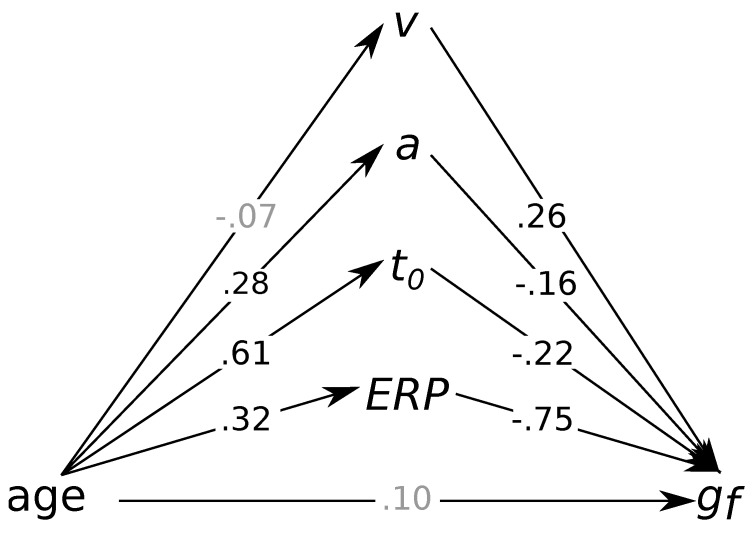
Multiple mediation model of the association between age differences and fluid intelligence. Standardized regression weights are shown next to paths with non-significant regression weights shaded in gray. *v* = drift rate; *a* = boundary separation; $t_{0}$ = non-decision time; ERP = event-related potential; $g_{f}$ = fluid intelligence. N=223.

To further explore which process-related parameter accounts for the association between age differences and fluid intelligence, we specified a multiple mediation model in which all four process parameters (i.e., diffusion model parameters and ERP latencies) were simultaneously introduced as mediators (see Figure 4). The model provided an acceptable account of the data, $\chi^{2}\left(394\right)=694.78$, p<0.001, CFI=0.83, RMSEA=0.06, 95% CI = [0.05; 0.07]. Both non-decision times, $\beta_{indirect}=-0.13$, p=0.046, 95% CI = [−0.26; −0.00], and ERP latencies, $\beta_{indirect}=-0.24$, p<0.001, 95% CI = [−0.36; −0.12], were significant mediators of the association between age differences and fluid intelligence. Conversely, individual differences in drift rates and boundary separations did not mediate the association between age differences and fluid intelligence, all $\beta s_{indirect}\leq \left|.05\right|$, all ps≥0.071. After introducing these mediators, the direct effect of age differences on fluid intelligence was no longer distinguishable from zero, β=0.10, p=0.269, 95% CI = [−0.08; 0.27]. Subsequently fixing the direct path to zero did not impair model fit, $\chi_{LLR}^{2}\left(1\right)=1.35$, p=0.246, suggesting that the association between and differences and fluid intelligence was fully mediated by individual differences in non-decision times and ERP latencies.

We also explored whether non-decision times and ERP latencies mediated the association between age differences and fluid intelligence jointly or independently. For this purpose, we constructed a model with a hierarchical higher-order factor loading onto both latent variables, which we introduced as the sole mediating variable of the association between age differences and fluid intelligence. Because the residual variance of the fluid intelligence factor was negative, we fixed this variance to zero. The resulting model provided an acceptable account of the data, $\chi^{2}\left(216\right)=360.05$, p<0.001, CFI=0.86, RMSEA=0.06, 95% CI = [0.05; 0.06]. This hierarchical factor (reflecting the shared variance between the latent non-decision time and ERP latency variables) significantly mediated the association between age differences and fluid intelligence, $\beta_{indirect}=-0.78$, p<0.001, 95% CI = [−1.00; −0.41], which suggests that individual differences in non-decision times and ERP latencies jointly accounted for the relationship between age differences and fluid intelligence. Surprisingly, we found some evidence for a suppressor effect, as the association between age differences and fluid intelligence became positive once individual differences in the hierarchical factor reflecting the shared variance between non-decision times and ERP latencies were accounted for, r=0.45, p=0.012, 95% CI = [0.11; 0.80]. This suppressor effect should, however, be interpreted with caution, because its estimation relies on model specifications resulting from the Heywood case (Heywood and Filon 1931) as described above. Fixing the direct path to zero resolved the Heywood case but significantly impaired model fit as indicated by larger AIC and BIC values, $\Delta_{AIC}=12,\Delta_{BIC}=12$. This suggests that the direct and indirect paths together fully explained individual differences in fluid intelligence and that the suppressor effect may be more than just a fluke in the data.

### 3.3. Do Age Differences Account for the Association between Process-Related Parameters and Fluid Intelligence?

Finally, we investigated whether age differences accounted for any of the association between process-related parameters and fluid intelligence. Although age did not explain any of the covariance between reaction times and fluid intelligence, it might still be possible that age differences give rise to some parts of the associations between diffusion model parameters and/or ERP latencies with intelligence.

**Figure 5:**
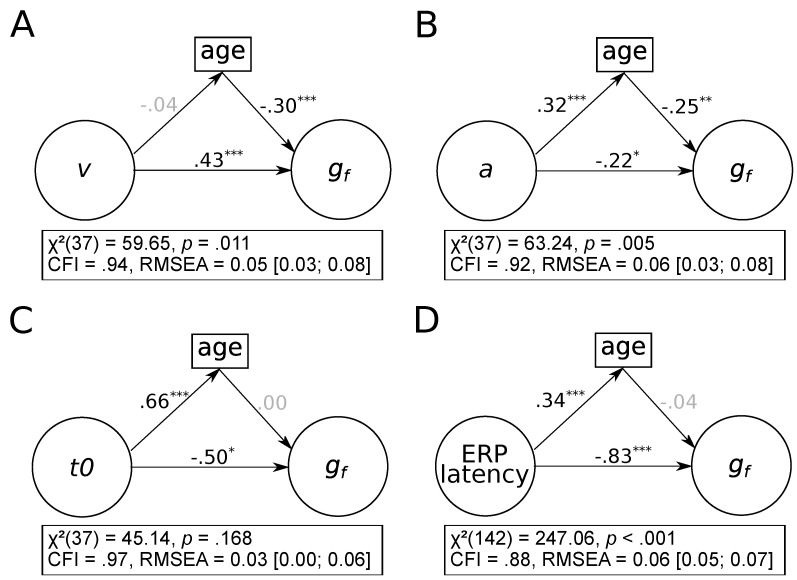
Latent mediation models for each of the four process-related parameters. (**A**) Drift rate. (**B**) Boundary separation. (**C**) Non-decision time. (**D**) ERP latency. Standardized regression weights are shown next to paths with non-significant regression weights shaded in gray. *v* = drift rate; *a* = boundary separation; $t_{0}$ = non-decision time; ERP = event-related potential; $g_{f}$ = fluid intelligence. N=223.

As illustrated in Figure 5 (p. 13), age differences did not account for much of the covariance between process-related parameters and fluid intelligence. Only the association between boundary separation and fluid abilities was partly mediated by age differences (see Figure 5B), $\beta_{indirect}=-0.08$, ps=0.007, 95% CI = [−0.14; −0.02]. For the other three process-related parameters (see Figure 5A for drift rates, Figure 5C for non-decision times, and Figure 5D for ERP latencies), age differences did not account for their association with fluid intelligence, all $\beta s_{indirect}\leq 0.01$, all ps≥0.559.

## 4. Discussion

The current study investigated which processes-related parameters may explain the finding that individual differences in processing speed fully mediate the association between age differences and fluid intelligence. We used a neurocognitive psychometrics account of mental speed to decompose the association between age differences and fluid intelligence and shed light on the question whether the processes giving rise to the association between age differences and fluid intelligence can be conceived of as a cause or an indicator of cognitive aging.

We replicated the finding that a large proportion of the age-related variance in fluid intelligence is shared with measures of processing speed (Verhaeghen and Salthouse 1997). Once this shared variance was accounted for, the association between age and fluid intelligence was no longer different from zero. We even found some tentative evidence for a suppressor effect in the way that the association between age differences and fluid intelligence became positive once individual differences in a joint factor of non-decision times and ERP latencies was accounted for. In addition, we also found that age did not account for any of the covariance between reaction times and fluid intelligence, which replicates previous research (Bors and Forrin 1995) and suggests that the association between processing speed and intelligence cannot be accounted for by age differences as a confounding variable.

### 4.1. Insights from a Neurocognitive Psychometrics Account of Mental Speed

When we decomposed the stream of information-processing using a neurocognitive psychometrics account, we found that both non-decision times and ERP latencies mediated parts of the association between age difference and fluid intelligence, whereas drift rate and boundary separation parameters did not. Surprisingly, drift rates were not related to age differences, as older participants did not show a lower velocity of evidence accumulation. These results are in line with previous research reporting consistent associations between age differences and non-decision times as well as decision boundaries, but inconsistent associations with drift rates (Dully et al. 2018).

Our results lead to two important conclusions: First, some of the covariance between age and fluid intelligence could be explained by individual differences in the speed of non-decisional processes such as encoding, memory retrieval, response preparation, and response execution. Because the non-decision time parameter encompasses the speed of both encoding- and response-related processes, individual differences in the speed of any (or all) of these processes may underlie the mediation. Hence, the age-related variance in non-decision times may reflect individual differences in brain properties prone to be affected by age-related neurodegenerative processes that result in an overall slowing of processing speed, supporting the idea that a general neurodegenerative process might account for the phenomenon that individual differences in processing speed mediate the association between age differences and fluid intelligence (Birren 1974; Hartley 2006). However, it is as of yet unclear why a general neurodegenerative process would not also consistently affect the speed of evidence accumulation captured in drift rates, unless age-related changes in drift rates only begin to occur at the age of 60 or older or unless the speed of evidence accumulation is in some way protected against general neurodegenerative processes. Alternatively, the age-related variance in non-decision times may reflect individual differences in the speed of visual encoding, which has been suggested to play an integral part in fluid intelligence (Sternberg 1977). What challenges this interpretation is the result that in our study latencies of ERP components occurring earlier in the stream of processing (i.e., N1 and P1) were not associated with age differences, all rs≤0.04. Nevertheless, latencies of very early sensory processing components, which cannot be easily assessed in the current study due to the relative complexity of the experimental tasks, may still be associated with age differences and the age-related variance in fluid intelligence. Finally, the association between age differences and non-decision times may also reflect individual differences in the speed of response-related processes such as response preparation and execution. However, from a theoretical point of view, it is unlikely that individual differences in these response-related processes give rise to intelligence differences. Therefore, they may reflect age-related differences in certain anterior brain regions associated with motor planning and response preparation that are also reflected in ERP latencies occurring later in the stream of information-processing such as the P3. This interpretation is supported by previous cross-sectional findings showing that anterior brain regions such as the prefrontal cortex follow a last-in-first-out pattern (Oschwald et al. 2019; Sowell et al. 2004) and play a substantial role in response preparation processes (Lee et al. 1999; Picard and Strick 1996). Finally, this association may reflect that participants’ performance on the intelligence test was affected by time restrictions, as older participants may have needed more time to check response options and may therefore have had less time to solve subsequent problems. Taken together, the fact that the association between age differences and fluid intelligence could be partly attributed to individual differences in the speed of non-decisional processes remains a conundrum that warrants further research.

Second, we found that individual differences in ERP latencies associated with higher-order attentional processes such as memory updating (Polich 2007), cognitive control (Folstein and Van Petten 2008), and attention (Folstein and Van Petten 2008) explained another part of the covariance between age and fluid intelligence. Latencies of these ERP components were negatively related to fluid intelligence and positively related to age. In other words, smarter individuals showed a greater neural processing speed during stages of higher-order information-processing, whereas older individuals showed a lower neural processing speed during these stages of information-processing. These results replicate previous studies that reported slower N2 and P3 latencies in older adults (Friedman 2012; Polich 1996).

All in all, our results suggest that the association between age differences and fluid intelligence may be explained by individual differences in the speed of non-decisional processes such as encoding, memory retrieval, response preparation, and response execution, and the speed of higher-order attentional processes. Because both parameters jointly accounted for the association between age differences and fluid intelligence, age-related differences in both parameters may reflect age-related differences in anterior brain regions associated with response planning that are prone to be affected by age-related changes. Taken together, our results paint the picture of a multifactorially determined relationship between age differences and fluid intelligence.

### 4.2. The Association between Processing Speed and Fluid Intelligence Cannot Be Accounted for by Age Differences

We replicated the finding that age differences did not account for correlation between reaction times and fluid intelligence (Bors and Forrin 1995) and could show that this also held true for the relationships of drift rates and ERP latencies with fluid intelligence. This is fairly important, because it demonstrates that it is unlikely that the previously reported associations of drift rates and ERP latencies with intelligence (Bazana and Stelmack 2002; McGarry-Roberts et al. 1992; Ratcliff et al. 2010; Schmiedek et al. 2007; Schmitz and Wilhelm 2016; Schmitz et al. 2018; Schubert et al. 2015; Troche et al. 2009; Ratcliff et al. 2011; Troche et al. 2015; Schubert et al. 2017; Schubert et al. 2019) can be attributed to age differences as a confounding factor. Instead, our results suggest that these associations are largely invariant with regard to age, although our results do not rule out the possibility that the form and strength of these associations might change across the lifespan. Conversely, at least some part of the correlations of boundary separation with fluid intelligence could be accounted for by age differences in the present study, which may explain inconsistencies in the association between this diffusion model parameter and cognitive ability measures in previous studies due to sample heterogeneity (Ratcliff et al. 2010; Schmiedek et al. 2007; Ratcliff et al. 2011).

### 4.3. Limitations

Some aspects of our study limit the conclusions we can draw from the results. First, only participants in an age range from 18 to 61 years were included in the present study, which limits any generalizations to old age. Although both cross-sectional and longitudinal studies have suggested that age differences in processing speed can be already observed in early adulthood and that processing speed continuously declines across the lifespan (Hartshorne and Germine 2015; Salthouse 2009; Schaie 2005), we do not yet know if the underlying cognitive processes behave in the same way, especially in old age.

Second, our conclusions were drawn based on cross-sectional data and may therefore be confounded with cohort effects. An increase in the proportion in cognitively demanding careers, for example, may have positively affected younger participants’ processing speed as well as their fluid intelligence. Future studies should therefore extend the neurocognitive psychometrics account presented in the current study to longitudinal data sets to disentangle age from cohort differences in mediating neurocognitive processes.

Third, because we only analyzed cross-sectional data, neither our results nor our conclusions can be generalized to questions of age-related cognitive change. In particular, the fact that measures of processing speed fully mediated the cross-sectional association between age and fluid intelligence does not imply that age-related changes in processing speed also explain age-related changes in fluid intelligence. It has often been observed that processing speed appears to be a stronger mediator of age effects on cognitive abilities in cross-sectional data than in longitudinal data (Zimprich and Martin 2002). Lindenberger et al. (2011) have demonstrated that one of the reasons cross-sectional mediation analyses tend to overestimate correlated developmental change is that strong mean age trends in the mediator contribute to cross-sectional age effects. Because individual differences in ERP latencies and non-decision times shared about 12 and 39 percent of variance with age differences, respectively, this may have contributed to an overestimation of the mediation effect that prohibits any generalization of our conclusions to correlated changes in processing speed and fluid intelligence.

Fourth, our conclusions rest entirely on the validity of the diffusion model. The diffusion model has been extensively validated (Lerche and Voss 2019; Voss et al. 2004) and is widely used in individual differences research (Frischkorn and Schubert 2018). Nevertheless, it is hard to explain why an association between age and non-decision times is consistently found across different studies (Dully et al. 2018), while associations between age and the speed of early visual processing as measured with the ERP are often inconsistent and typically small (De Sanctis et al. 2008; Friedman 2012). If the association between the age-related variance in fluid intelligence and non-decision times reflects a general neurodegenerative process, however, it is implausible why this process does not also consistently affect the speed of evidence accumulation captured in drift rates. Because our results are largely consistent with the literature, it is unlikely that our results were overly affected by our choice of fairly simply decision tasks. Nonetheless, it may be worthwhile to further validate the diffusion model by combining experimental and correlational approaches.

Fifth, the role of non-decisional processes in explaining the association between age and fluid intelligence decline may have been overestimated in the present study because fluid intelligence was measured with the Berlin Intelligence Structure test, which contains varying time limits for each of its subtests. In particular, older participants may have performed worse in some subtests with a rather strict speed limit due to lower encoding and motor response speed, resulting in a smaller number of tackled and subsequently solved items that would then have been interpreted as reflecting lower fluid intelligence. It is therefore possible that non-decision times mediated the association between age and fluid intelligence because older participants actually performed worse in the intelligence test due to lower encoding and motor response speeds. Hence, it would be imperative to replicate our results using a power test that does not rely on speed constraints.

## 5. Conclusions

We used a neurocognitive psychometrics approach to investigate which process-related parameter may explain the finding that individual differences in processing speed fully mediate the association between age differences and fluid intelligence. Combining diffusion modeling with chronometric analyses of the ERP, we found that the association between age and fluid intelligence could be fully explained by individual differences in non-decision times and latencies of ERP components associated with higher-order cognitive processes. Because both parameters jointly accounted for the association between age differences and fluid intelligence, age-related differences in both parameters may reflect age-related differences in anterior brain regions associated with response planning that are prone to be affected by age-related changes. Conversely, age differences did not account for the associations between measures of processing speed and intelligence. Taken together, these results suggest that the relationship between age differences and fluid intelligence is multifactorially determined.

## Figures and Tables

**Table 1:** Mean (SDs in parentheses) accuracies (ACC), reaction times (RT), diffusion model parameters, and event-related potential (ERP) latencies for each condition of the experimental tasks.

	ACC	RT	v	a	t0	P2 Latency	N2 latency	P3 Latency
Sternberg task								
S3	0.98 (0.02)	799.30 (203.15)	2.93 (0.91)	1.85 (0.74)	0.46 (0.12)	237.41 (40.13)	253.49 (50.21)	362.62 (91.23)
S5	0.97 (0.03)	961.99 (279.98)	2.44 (0.98)	2.00 (0.89)	0.53 (0.16)	237.54 (35.41)	253.02 (50.27)	387.50 (94.89)
Posner task								
PI	0.99 (0.02)	629.79 (100.31)	4.25 (1.50)	1.59 (0.77)	0.43 (0.08)	220.63 (37.97)	246.80 (42.80)	411.03 (94.46)
NI	0.98 (0.02)	707.39 (113.15)	3.26 (1.07)	1.52 (0.47)	0.46 (0.07)	225.03 (30.51)	244.78 (36.63)	418.36 (88.49)
